# A comparison of off- and on-pump beating-heart coronary artery bypass surgery on long-term cardiovascular events

**DOI:** 10.5830/CVJA-2016-049

**Published:** 2017

**Authors:** Orcun Gurbuz, Gencehan Kumtepe, Abdulkadir Ercan, Atıf Yolgosteren, Hakan Ozkan, Ilker Hasan Karal, Serdar Ener

**Affiliations:** Department of Cardiovascular Surgery, Faculty of Medicine, Balikesir University, Balikesir, Turkey; Department of Cardiovascular Surgery, Faculty of Medicine, Balikesir University, Balikesir, Turkey; Department of Cardiovascular Surgery, Faculty of Medicine, Balikesir University, Balikesir, Turkey; Department of Cardiovascular Surgery, Faculty of Medicine, Uludag University, Bursa, Turkey; Department Of Cardiology, Faculty of Medicine, Bahcesehir University, Istanbul, Turkey; Department of Cardiovascular Surgery, Samsun Hospital for Education and Research, Ilkadim, Samsun, Turkey; Department of Cardiovascular Surgery, Doruk Yıldırım Hospital, Bursa, Turkey

**Keywords:** major cardiovascular event, off-pump coronary artery bypass grafting, on-pump beating heart

## Abstract

**Objective:**

Our aim was to compare short-term outcomes and long-term major adverse cardiovascular event (MACE)-free survival and independent predictors of long-term MACE after off-pump (OPCAB) versus on-pump beating-heart (ONBHCAB) coronary artery bypass grafting (CABG).

**Methods:**

We retrospectively reviewed data of all consecutive patients who underwent elective CABG, performed by the same surgeon, from January 2003 to October 2009. A propensity score analysis was carried out to adjust for baseline characteristics and a total of 398 patients were included: ONBHCAB (n = 181), OPCAB (n = 217).

**Results:**

OPCAB was associated with significantly shorter ventilation times (p < 0.001), intensive care unit stay (p < 0.001) and hospital stay (p < 0.001). The total blood loss was significantly more in the ONBHCAB group (p < 0.001), and accordingly, the number of transfused blood units was significantly lower in the OPCAB group (p < 0.001). Incidence of peri-operative renal complications were significantly higher in the ONBHCAB group (p = 0.004). The OPCAB group showed significantly lower long-term MACE-free survival (p = 0.029). The mean number of transfused blood units was the only independent predictor of MACE (HR: 1.218, 95% CI: 1.089–1.361; p = 0.001).

**Conclusion:**

OPCAB provided better long-term MACE-free survival compared with ONBHCAB. Fewer units of blood transfused following OPCAB surgery may have been the main reason for this result.

## Objectives

The adverse effects of cardiopulmonary bypass (CPB), aortic cross-clamping and cardioplegic arrest have brought about growing interest in off-pump coronary artery bypass surgery (OPCAB) since the mid 1990s, as a strategy to protect high-risk patients from complications.^1^ Although OPCAB has advantages,^2^ it also carries some risks, such as intra-operative low cardiac output and inadequate revascularisation.^3^,^4^ Therefore, the debate over the optimal method of revascularisation continues

In recent years, as an alternative to both techniques, the on-pump beating-heart coronary artery bypass grafting (ONBHCAB) technique has gained acceptance in order to eliminate the harmful effects of cross-clamping, cardioplegia and unloading the heart, and it preserves both native coronary blood flow and cardiac output during surgery.^5^-^7^ Although a metaanalysis revealed better short-term outcomes and late survival rates following ONBHCAB compared with conventional CABG (CCAB),^7^ studies comparing the outcomes of ONBHCAB and OPCAB techniques in a similar patient population are lacking. Therefore we aimed to compare the short-term outcomes and long-term major adverse cardiovascular event (MACE)-free survival after ONBHCAB versus OPCAB in a matched population.

## Methods

The research was conducted according to the principles of the Declaration of Helsinki, and ethical approval was granted by the local research ethics committee. In this retrospective study, we reviewed data for all patients who underwent isolated firsttime elective coronary bypass surgery at Uludag University Faculty of Medicine Hospital and Bursa Medical Park Hospital between January 2003 and October 2009. The same surgeon performed the ONBHCAB and OPCAB techniques. There were no described selection criteria between the two techniques.

Exclusion criteria were as follows: critical pre-operative state [need for inotropic drug support or intra-aortic balloon pumping (IABP), acute renal failure, requiring respiratory support, history of cardiopulmonary resuscitation in the pre-operative period], myocardial infarction (MI) within three weeks [cardiac troponin I (cTnI) > 0.01 ng/ml], patients who underwent single-vessel CABG, and cases that were converted from OPCAB to ONBHCAB (12 of 339 cases, 3.5%) or ONBHCAB to conventional CABG [10 of 443 cases (2.2%)] intra-operatively.

Finally, 760 patients were divided into two groups: ONBHCAB (group 1) or OPCAB (group 2). To adjust for baseline differences in parameters between the groups, a propensity score analysis was carried out and a total of 398 patients were included: ONBHCAB (n = 181), OPCAB (n = 217).

Patients’ pre-operative characteristics, such as age and gender, smoking status, hypertension, diabetes mellitus (DM), dyslipidaemia, obesity (body mass index > 30 kg/m^2^), chronic obstructive pulmonary disease (COPD), history of stroke, peripheral vascular disease (PVD), history of myocardial infarction (MI), unstable angina pectoris (USAP), EuroSCORE (European System for Cardiac Operative Risk Evaluation) risk score, left ventricular dysfunction, history of percutaneous coronory intervention (PCI), number of diseased vessels, and the presence of left main coronary artery (LMCA) stenosis were recorded.

## Definitions

Vessel disease was defined as stenosis of more than 50% of the major epicardial coronary arteries. Estimated creatinine clearance (CrCl) rate was calculated using the Cockcroft–Gault formula: CrCl (ml/min) = [(140–age) × weight (kg)]/[serum creatinine (mg/dl) × 72] × 0.85 for women, from baseline blood samples. PVD was defined as a stenosis of 50% or more affecting any non-coronary vasculature.

Left ventricular dysfunction was defined as moderate [ejection fraction (EF) 0.30–0.49%] or severe (EF < 0.30%). Complete revascularisation was defined as treatment of all major coronary arteries [left anterior descending (LAD), circumflex (Cx) and right coronary artery (RCA)] ≥ 50% diameter stenosis.

Total blood loss was defined as the sum of the mediastinal and chest tube drainage in the first 48 hours. Consumed units of red blood cells (RBC) was defined as the sum of the blood units used during the hospital stay. Any inotropic support started in the peri-operative period, even low doses of dopamine infusion due to haemodynamical instability, was determined as peri-operative need for inotropic support. Peri-operative MI was defined as cTnI > 5 μg/l during the hospital stay with new ECG change or echocardiographic evidence of new regional wall motion abnormality.^8^

Renal complication was defined as at least 100% increase in basal serum creatinine level. Pulmonary complication was defined as pleural effusion, atelectasis, phrenic nerve paralysis, diaphragmatic dysfunction, pneumonia, acute respiratory distress syndrome, pneumothorax or chylothorax. Neurological complication was defined as any new transient ischaemic attack (TIA), stroke or encephalopathy occurring in the peri-operative period.

Early rehospitalisation was defined as any hospitalisation due to CABG-related complications (such as sternal dehiscence, mediastinitis) or cardiovascular problems (such as MI, congestive heart failure, rhythm disturbance, neurological complications, pulmonary embolism). Early re-operation was defined as re-operation due to bleeding or cardiac tamponade and graft failure.

## Surgical procedures

All procedures were performed by the same surgeon, who made the decision to perform OPCAB or ONBHCAB surgery. Classic median sternotomy, left internal thoracic artery (LIMA) harvesting and other conduit preparations were performed according to a standard technique. In patients undergoing OPCAB, heparin was administered to keep the activated clotting time (ACT) greater than 300 seconds.

Distal anastomoses were performed by end-to-side or side-toside techniques with a running 7/0 Prolene suture, using a local myocardial stabiliser (Octopus, Medtronic Inc, Minneapolis, MN, US). Proximal coronary clamping of all target vessels was performed with Mueller atraumatic vascular clamps (0.5 Newton); distal occlusion was never performed. Insufflation of filtered room air (< 5 l/min) was used to provide better visibility during anastomosis. During distal anastomosis and reperfusion, 2 ml/kg 20% mannitol was administered. All proximal anastomoses were performed under single side clamping using 6/0 prolene sutures.

At the end of surgery, heparin was neutralised with protamine, ensuring that the ACT was between 150 and 180 seconds. In the early postoperative period (6–8 hours), low molecularweight heparin and 100 mg acetylsalicylic acid were commenced routinely.

In patients undergoing ONBHCAB, heparin was administered to keep the ACT above 450 seconds. CPB was established with an ascending aortic arterial cannula and a right atrial two-stage venous cannula, using a membrane oxygenator and a roller pump. All patients were cooled to 32–34°C. Mean arterial blood pressure was maintained in the range of 60–90 mmHg. Distal anastomoses were performed by end-to-side or side-to-side techniques with a running 7/0 prolene suture, using a myocardial stabiliser device (Octopus, Medtronic Inc, Minneapolis, MN, US). Proximal anastomoses were performed using a 6/0 prolene suture during the heating period with the assistance of an ascending aortic side-clamp. After the completion of CPB and cannula removal, heparin was neutralised with protamine, providing an ACT < 150 seconds. Acetylsalicylic acid at a dose of 100 mg and low molecular-weight heparin was initiated at the postoperative 24th hour.

The primary endpoint of this study was to compare the early and long-term MACE rates, defined as cardiac related or sudden death, MI, the need for repeat revascularisation, and stroke following ONBHCAB versus OPCAB. The secondary endpoint was to identify independent predictors of long-term MACE in these groups of patient. Long-term follow up was obtained through out-patient clinic visits, hospital records and phone calls. All-cause mortality (patient death reported by patients’ relatives or hospital records) and MACE were determined.

## Statistical analysis

Continuous variables are expressed as mean ± standard deviation. Categorical variables are expressed as percentages. A propensity score analysis was carried out to control selection based on the baseline variables. The Mann–Whitney U-test was used to compare non-parametric continuous variables, the Student’s t-test was used to compare parametric continuous variables, and the chi-squared test was used to compare categorical variables.

Cumulative survival curves for long-term MACE were constructed using the Kaplan–Meier method, whereas differences between the groups were evaluated with log-rank tests. Multivariate logistic regression analysis was used to identify the independent predictors of MACE. All variables showing significance values (p < 0.05) on univariate analysis were included in the multivariate model. The association between variables was tested using Spearman’s or Pearson’s correlation coefficient. Two-tailed p-values < 0.05 were considered significant and the confidence interval (CI) was 95%. All statistical analyses were conducted using the Statistical Package for Social Sciences (SPSS) program (version 15.0, SPSS, Chicago, Illinois, USA).

## Results

Pre-operative patient characteristics for the ONBHCAB and OPCAB groups before and after matching are listed in Table 1. Peri-operative and early postoperative characteristics of the propensity-matched patients are shown in Table 2.

**Table 1:** Baseline characteristics of the study groups

**	*Overall (n = 760)*			*Propensity-matched patients (n = 398)*		
	ONBHCAB	OPCAB		ONBHCAB	OPCAB	
Characteristics	(n = 327)	(n = 433)	p-value	(n = 181)	(n = 217)	p-value
Age (years)	63.37 ± 9.65	59.7 ± 9.08	< 0.001	61.17 ± 9.02	60.16 ± 8.8	0.3
Males	255 (78)	359 (82.9)	0.08	147 (81.2)	179 (82.5)	0.74
Euroscore	3.9 ± 2.41	2.79 ± 1.99	< 0.001	3.06 ± 2.13	2.83 ± 2.1	0.17
Obesity (BMI ≥ 30 kg/m^2^)	93 (28.4)	80 (18.5)	0.001	53 (29.3)	48 (22.1)	0.1
Current smoker	142 (43.4)	234 (54)	0.004	85 (47)	113 (52.1)	0.31
Hypertension	172 (52.6)	245 (56.6)	0.27	95 (52.5)	126 (58.1)	0.26
Dyslipidaemia	114 (34.9)	181 (41.8)	0.05	62 (34.3)	89 (41)	0.16
Diabetes mellitus	102 (31.2)	125 (28.9)	0.48	58 (32)	64 (29.5)	0.58
NIDDM	74 (22.6)	98 (22.6)	0.99	44 (24.3)	46 (21.2)	0.46
IDDM	28 (8.6)	27 (6.2)	0.22	14 (7.7)	18 (8.3)	0.83
USAP	97 (29.7)	138 (31.9)	0.51	46 (25.4)cc	74 (34.1)	0.6
COPD	36 (11)	12 (2.8)	< 0.001	14 (7.7)	9 (4.1)	0.12
CrCl	103.3 ± 34.13	101.06 ± 38.9	0.04	102.35 ± 40.6	101.19 ± 30.58	0.21
Stroke	7 (2.1)	14 (3.2)	0.36	4 (2.2)	7 (3.2)	0.54
TIA	7 (2.1)	8 (1.8)	0.77	4 (2.2)	4 (1.8)	0.79
Peripheral vascular disease	65 (19.9)	30 (6.9)	< 0.001	29 (16)	25 (11.5)	0.19
History of MI	127 (38.8)	189 (43.6)	0.18	53 (29.3)	81 (37.3)	0.91
Normal LV function	213 (65.1)	316 (73)	0.02	129 (71.3)	162 (74.7)	0.45
Impaired LV function	114 (34.9)	117 (27)	0.02	52 (28.7)	55 (25.3)	0.45
Moderate LV function	96 (29.4)	104 (24)	0.09	44 (24.3)	49 (22.6)	0.68
Poor LV function	18 (5.5)	13 (3)	0.08	8 (4.4)	6 (2.8)	0.37
Previous PCI	37 (11.3)	16 (3.7)	< 0.001	15 (8.3)	9 (4.1)	0.54
Number of diseased vesssels	2.69 ± 0.56	2.62 ± 0.54	0.01	2.69 ± 0.56	2.64 ± 0.53	0.12
LMCA	59 (18)	45 (10.4)	0.002	30 (16.6)	24 (11.1)	0.11
Threevessel disease	246 (75.2)	285 (65.8)	0.005	135 (74.6)	146 (67.3)	0.12

*Statistically significant difference. Values are presented as mean ± standard deviation or number (%), where appropriate.

BMI, body mass index; CrCL, creatinine clearance; CAD, coronary artery disease; COPD, chronic obstructive pulmonary disease; IDDM, insulin-dependent diabetes mellitus; LMCA, left main coronary artery; LV, left ventricle MI, myocardial infarction; NIDDM, non-insulin-dependent diabetes mellitus; ONBHCAB, on-pump beating heart coronary artery bypass surgery; OPCAB, off-pump coronary artery bypass surgery; PCI, percutaneous coronary intervention; PVD, peripheral vascular disease; TIA, transient ischaemic attack; USAP, unstable angina pectoris.

**Table 2:** Operative and early postoperative characteristics of the propensity matched patients

**	*ONBHCAB*	*OPCAB*	**
*Characteristics*	*(n = 181)*	*(n = 217)*	*p-value*
Number of distal anastomoses	3.44 ± 0.85	2.84 ± 0.81	< 0.001*
Incomplete revascularisation	41 (22.7)	55 (25.3)	0.53
Grafted coronary artery			
LAD	1.64 ± 0.5	1.39 ± 0.55	< 0.001*
Cx	0.96 ± 0.58	0.71 ± 0.56	0.003*
RCA	0.82 ± 0.58	0.79 ± 0.59	0.05
CPB time (min)	70 ± 35		
Duration of ventilation (h)	11.28 ± 38.7	5.97 ± 4.01	< 0.001*
Duration in intensive care unit (h)	28.27 ± 63.9	25.48 ± 36.1	< 0.001*
Total blood loss (ml)	507.6 ± 296.8	341.9 ± 190.4	< 0.001*
RBC (unit)	1.17 ± 1.57	0.2 ± 0.58	< 0.001*
Peri-operative inotropic agent	8 (4.4)	6 (2.8)	0.37
Peri-operative IABP required	4 (2.2)	2 (0.9)	0.29
Peri-operative MI	11 (6.1)	13 (6)	0.97
Peri-operative AF	21 (11.6)	28 (12.9)	0.69
Neurological complication	11 (6.1)	6 (2.8)	0.10
Encephalopathy	4 (2.2)	1 (0.5)	0.11
TIA	6 (3.3)	4 (1.8)	0.35
Stroke	1 (0.6)	1 (0.5)	0.89
Pulmonary complications	11 (6.1)	7 (3.2)	0.17
Renal complications	24 (13.3)	11 (5.1)	0.004*
Mediastinitis	2 (1.1)	1 (0.5)	0.95
Duration of hospital stay (days)	5.96 ± 3.51	5.41 ± 4.38	< 0.001*
Early re-operation	5 (2.8)	3 (1.4)	0.33
Early rehospitalisation (< 30 days)	13 (7.2)	14 (6.5)	0.92
Hospital mortality (< 30 days)	3 (1.7)	6 (2.8)	0.46

*Statistically significant difference. Values are presented as mean ± standard deviation or number (%), where appropriate.

AF, atrial fibrillation; LAD, left anterior descending artery; Cx, circumflex coronary artery; IABP, intra-aortic balloon pump; MI, myocardial infarction; ONBHCAB, on-pump beating heart coronary artery bypass surgery; OPCAB, off-pump coronary artery bypass surgery; RBC, red blood cell; RCA, right coronary artery; TIA, transient ischaemic attack.

The average number of distal anastomoses per patient was significantly higher in the ONBHCAB group (p < 0.001). The OPCAB group showed fewer grafted LAD and Cx territories (p < 0.001, p = 0.003, respectively). However, there was no significant difference between the groups in terms of incomplete revascularisation rate.

Mean duration of CPB was 70 ± 35 minutes in the ONBHCAB group. OPCAB was associated with significantly shorter mean ventilation times (p < 0.001), mean lengths of intensive care unit stay (p < 0.001) and duration of hospital stay (p < 0.001). The total blood loss was significantly more in the ONBHCAB group (p < 0.001). Accordingly, the mean number of transfused RBC units was significantly lower in the OPCAB group (p < 0.001). Renal complications were significantly higher in the ONBHCAB group (p = 0.004). Postoperative characteristics of the groups were similar regarding early re-operation and rehospitalisation, in-hospital mortality rates, mediastinitis, pulmonary and neurological complications, peri-operative atrial fibrillation (AF) frequency, peri-operative MI, and the need for inotropic or IABP support.

**Fig. 1.**
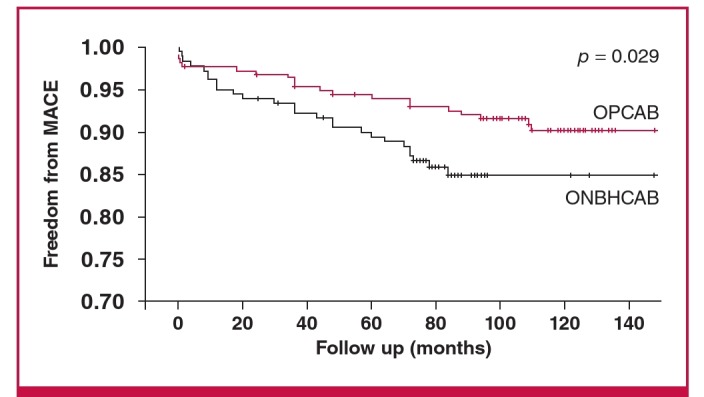
Kaplan–Meier estimates in the propensity-matched populations. Freedom from major adverse cardiovascular events (MACE). Red lines indicate OPCAB group, black lines indicate ONBHCAB group (p = 0.029 by the log-rank test).

**Fig. 2.**
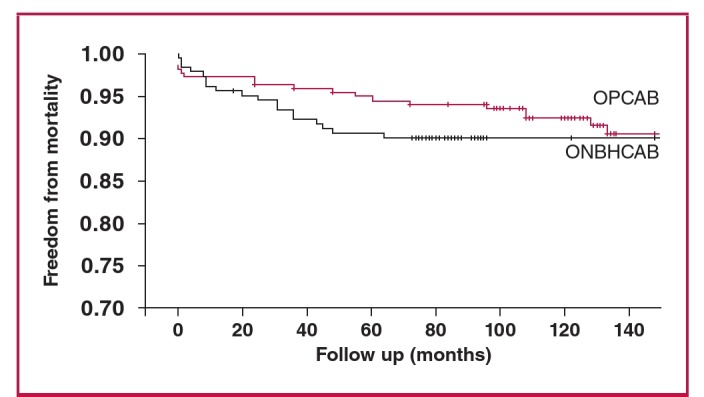
Kaplan–Meier estimates in the propensity-matched populations. Freedom from mortality. Red lines indicate OPCAB group, black lines indicate ONBHCAB group (p = 0.16 by the log-rank test).

Kaplan–Meier analysis of freedom from MACE revealed significantly lower event-free survival rates in the OPCAB group (ONBHCAB, 84.9%; OPCAB, 90.3%; p = 0.029 by the log-rank test) (Fig. 1). Kaplan–Meier analysis of freedom from mortality revealed no significant difference between the two groups (ONBHCAB, 90%; OPCAB, 90.5%; p = 0.16 by the log-rank test) (Fig. 2). In the multivariable Cox proportional hazard model, the mean number of transfused RBC units was the only independent significant predictor of MACE (Table 3).

**Table 3:** Cox proportional hazard model for MACE at the long-term follow up of propensity-matched patients

**	*Univariate analysis*			*Multivariate analysis*		
*Characteristics*	*HR*	*95% CI*	*p-value*	*HR*	*HR 95% CI*	*p-value*
Pre-operative characteristics						
Age	1.026	0.992–1.061	0.14			
Males	1.519	0.77–2.998	0.22		
EuroSCORE	1.299	1.140–1.479	< 0.001*	1.051	0.873–1.265	0.59
Obesity (BMI ≥ 30 kg/m^2^)	1.891	1.006–3.557	0.048*	1.681	0.812–3.481	0.16
CrCl	0.999	0.991–1.007	0.75			
COPD	2.534	0.999–6.425	0.05	1.102	0.317–3.826	0.87
Diabetes mellitus	1.297	0.705–2.389	0.4			
Cerebrovascular disease	1.439	0.446–4.641	0.54			
Peripheral vascular disease	2.335	1.183–4.609	0.01*	0.982	0.366–2.634	0.98
Previous MI	2.619	1.454–4.716	0.001*	1.764	0.808–3.851	0.15
Impaired LV function	1.951	1.074–3.542	0.02*	1.186	0.529–2.659	0.68
USAP	1.446	0.796–2.625	0.22			
Previous PCI	0.727	0.176–3.000	0.65			
Number of diseased vessels	1.806	0.923–3.534	0.08	1.435	0.664–3.099	0.35
Operative factors						
Number of distal anastomoses	0.921	0.658–1.288	0.63			
CPB time	1.005	0.991–1.019	0.51			
Total blood loss (ml)	1.001	1.000–1.002	0.18			
RBC (units)	1.328	1.141–1.545	< 0.001*	1.218	1.089–1.361	0.001*
Postoperative inotropic support	1.847	0.913–3.733	0.08	1.047	0.584–7.825	0.25
Peri-operative MI	2.764	1.170–6.530	0.02*	1.642	0.474–5.695	0.43
Renal complications	2.131	0.951–4.778	0.06	0.836	0.269–2.597	0.75
Neurological complications	2.554	0.914–7.135	0.07	2.137	0.584–7.825	0.25
Early rehospitalisation (< 30 days)	2.852	1.273–6.387	0.011*	1.256	0.410–3.851	0.69

*Statistically significant difference.

BMI, body mass index; CABG, coronary artery bypass graftıng; CI, confidence interval; CrCl, creatinine clearance; COPD, chronic obstructive pulmonary disease; DM, diabetes mellitus; HR, hazard ratio; LV, left ventricle; MI, myocardial infarction; ONBHCAB, on-pump beating heart coronary artery bypass surgery; OPCAB, off-pump coronary artery bypass surgery; PCI, percutaneous coronary intervention; RBC, red blood cell, USAP, unstable angina pectoris.

## Discussion

OPCAB has the potential to reduce several of the adverse effects of CCAB because of elimination of CPB, the harmful effects of cardioplegia, the cessation of coronary blood flow, and excessive aortic manipulation. Its superiority compared with CCAB in early or mid-term outcomes has therefore been reported by many authors.^9^-^12^ However, the risk of haemodynamic instability during surgery, causing incomplete revascularisation, especially in the hands of an inexperienced surgeon, remains the major limitation of this method.

A third method, ONBHCAB, reduces myocardial ischaemia, maintaining coronary blood flow, preserves haemodynamic stability with the use of CPB, and permits cardiac manipulation. Moreover, it also limits aortic manipulation and protects the heart from post-cardioplegic intimal damage, as well as OPCAB surgery. A recent meta-analysis showed that ONBHCAB was associated with significantly fewer peri-operative MIs and less IABP use, shorter CPB time, and lower total blood loss compared with CCAB. However, it was similar in terms of cerebrovascular events, renal dysfunction, pulmonary complications, re-operation due to bleeding, inotropic agent use, intensive care unit stay, hospital stay, ventilation time, number of anastomoses, early mortality and mid-term survival rates after CABG.^7^

Another recent meta-analysis revealed that OPCAB was associated with significantly fewer incidents of peri-operative low cardiac output (LCO) and renal dysfunction, less total blood loss, fewer RBC transfusions, shorter ventilation times and lengths of ICU or hospital stay, but similar rates of in-hospital or long-term mortality, peri-operative MIs and cerebrovascular accidents within the first 30 days compared with CCAB. Moreover, OPCAB was also associated with an increased risk of repeat revascularisation within the first month and significantly lower numbers of performed grafts.

Studies comparing the outcomes of OPCAB versus ONBHCAB are however limited.^5^ Therefore we evaluated early outcomes and long-term MACE rates of ONBHCAB versus OPCAB in a matched population. All patients were operated on by the same surgeon, therefore positioning of the heart and anastomosis techniques were typically similar between the groups, except for the use of CPB.

The need for inotropic agents or IABP were found to be similar between the groups. Moreover, no difference was detected in terms of incidence of peri-operative MI and AF between the two techniques. These findings indicate that both OPCAB and ONBHCAB techniques may cause similar myocardial damage, which may explain the blood loss during both procedures.

Stroke is generally considered the most important coronary surgery-related morbidity. Many meta-analyses have revealed that OPCAB is associated with short- and long-term benefits in stroke prevention, especially in higher-risk patients.^1^,^13^ By contrast, Moller and co-workers’ meta-analysis revealed no significant benefit of OPCAB compared with ONCAB regarding stroke.^14^

In our study, no significant difference was detected among the groups in terms of peri-operative stroke, TIA or encephalopathy. These findings revealed that the avoidance of aortic crossclamping may reduce embolic particles. The duration of ventilation, and ICU and hospital stays were significantly shorter in the OPCAB group, as in previous publications.15-17 The amount of drainage in the first 48 hours was significantly lower in the OPCAB group, therefore, the mean number of transfused RBC units was significantly lower in the OPCAB group. These findings may be explained by the well-known adverse effects of extracorporeal circulation and hypothermia on the coagulation system.^15^-^17^

Chaudhry’s meta-analysis^7^ revealed similar renal dysfunction after ONBHCAB in comparison with CCAB. In our study, despite similar pre-operative levels of EF, CrCl and perioperative LCO, the OPCAB group showed significantly lower renal complications than the ONBHCAB group. This finding supports a previous report indicating the independent negative effect of CPB on renal function.^18^

Two different meta-analyses of randomised trials reported a significantly lower number of distal anastomoses performed per patient following off-pump versus on-pump surgery.^7^,^19^ Similarly, in our study, the number of distal anastomoses per patient was significantly lower in the OPCAB group. However, in terms of functionally incomplete revascularisation, no difference was detected between the groups. It is clear that the advantage of haemodynamic stability of ONBHCAB made the surgeon feel more at ease than with OPCAB and he performed better anastomoses.

Two different meta-analyses of randomised trials revealed no significant differences between off-pump and on-pump CABG regarding all-cause mortality and MACE.^7^,^8^ Our study also revealed similar all-cause mortality rates between the groups, the OPCAB group showing a significantly better MACE-free period, including MI, PCI, redo CABG and stroke in the long term. Moreover, we found that the mean number of transfused RBC units was the only significant predictor of MACE following CABG. This was considered the main cause of the negative results in the ONBHCAB group.

It is clear that CPB is a challenge to the haematopoietic system due to haemodilution, significant shifts in intravascular volume, mechanical trauma to the blood cells and hypothermia, leading to increased transfusion of RBC or blood products.^2^,^13^,^15^,^16^ Transfusion of RBC as a risk factor for early mortality following CABG has been well established, whereas the effect of RBC transfusion on late mortality or MACE is less well described. A number of studies have shown the negative impact of RBC transfusion on early cardiovascular events and late mortality rates following cardiac surgery.^20^-^22^ RBC transfusion was also found to be associated with peri-operative MI following elective isolated OPCAB.^23^ Additionally, a recent report showed that low-risk patients had a significantly higher long-term mortality rate when receiving RBC following cardiac surgery, compared with patients who did not receive transfusions. This effect was not seen in high-risk patients, suggesting the negative impact of the use of blood was independent of other risk factors.^24^ RBC transfusion was also found to be associated with a strongly increased risk of both 30-day cardiovascular events and mortality in elective vascular surgery patients.^25^

The reasons for such a correlation between long-term cardiovascular events and blood transfusion are unclear but the pro-inflammatory properties of transfused RBC have been suggested as a potential explanation. It has been well established that inflammation plays a major role in all stages of atherogenesis. The role of inflammation in the pathogenesis of ischaemic stroke,^26^ MI and neo-intimal hyperplasia leading to in-stent restenosis27 or graft failure^28^ has also been described. Moreover, Fransen et al. showed that blood transfusion may potentialise the inflammatory effect of CPB.^29^ The combined effect of RBC transfusion and CPB may therefore aggravate atherosclerosis by stimulating the ongoing inflammatory process in patients with coronary artery disease.

The present study has some limitations, including its retrospective, non-randomised design and relatively small sample size. However, our population contained propensity-matched, homogeneous patients undergoing CABG surgery by the same surgeon, using the same technique, ONBHCAB. Therefore, other factors interacting with the frequency of MACE due to differences in surgical technique or patient demographics were excluded.

## Conclusion

Off-pump CABG provided better long-term MACE-free survival compared with on-pump beating-heart CABG. Decreased incidence of blood transfusion following OPCAB surgery may have been the main reason for this.
